# Design of a Nested Hollow-Core Anti-Resonant Fiber Sensor for Simultaneous Measurement of Temperature and Strain

**DOI:** 10.3390/s24237805

**Published:** 2024-12-06

**Authors:** Yueyu Xiao, Jiayao Cheng

**Affiliations:** 1 Key Laboratory of Specialty Fiber Optics and Optical Access Networks, Shanghai University, Shanghai 200444, China; chengjiayao036@shu.edu.cn; 2 Institute of Fiber Optics, Shanghai University, Shanghai 201800, China

**Keywords:** hollow-core anti-resonant fiber, dual-parameter sensing, temperature, strain

## Abstract

A highly sensitive sensor, which can detect the temperature and strain simultaneously, is proposed using a hollow-core anti-resonant fiber with composite nested tubes. The sensing fiber contains two kinds of nested tubes, and two different sensing mechanisms, the resonance coupling effect and the intermodal interference, are realized in the same section of a hollow-core anti-resonant fiber fully filled with ethanol. Five conjoined nested anti-resonant tubes are introduced to suppress the confinement loss of the higher-order mode LP_02_. One hybrid conjoined nested tube, which consists of a half-circular anti-resonant tube and a half-circular resonant tube, is introduced to induce a resonant coupling between the LP_02_ mode in the core and the dielectric mode in the nested resonant tubes. Numerical investigations demonstrate the shifts of the feature wavelengths of the resonance coupling effect, and the intermodal interference shows different velocities with temperature and strain, while a simultaneous measurement of temperature and strain can be realized with high sensitivities (3.36 nm/°C and −0.003 nm/με to temperature and strain, respectively). Since the sensor can be fabricated by full infiltration with liquid into the large-size core and cladding tubes of hollow-core anti-resonant fibers, and special post-processing, such as selective infiltration or coating, is notneeded. The proposed sensors based on hollow-core anti-resonant fibers with functional liquid infiltration provide a more efficient and versatile platform for the temperature and strain sensing.

## 1. Introduction

Fiber optical sensors have been widely used in the measurement of various physical parameters, such as temperature, humidity, force, etc. [1,2]. Temperature and strain are the basic state parameters of a subject, and the measurement of temperature and strain has received much attention in the fiber optical sensing area. Since silica is cross-sensitive to temperature and strain, several methods have been proposed to address this issue, including the single-parameter (temperature or strain) compensation and the dual-parameter interrogation. The former always makes sensors complicated and expensive [3], and the latter is more efficient because the temperature and strain can be obtained simultaneously. Various fiber sensors have been developed for the simultaneous measurement of temperature and strain, such as the cascaded structure of two fiber gratings [4,5], the combination of fiber Bragg gratings with hi-bi fiber loop mirrors or Mach–Zehnder interferometers [6,7], and multi-interferometric serial configurations [8,9]. However, owing to the lack of design flexibility of traditional solid-core fibers, the dual-parameter sensors always have a low sensitivity and a complicated structure.

Photonic crystal fibers (PCFs) contain axially aligned air channels, and the selective infiltration of liquid into the air holes of PCFs can greatly improve the sensitivity of fiber optical sensors. To address the cross-sensitive problem in PCFs, Liang et al. proposed a double-filled photonic crystal fiber in 2013, and the simultaneous measurement of temperature and force can be obtained [10]. In 2016, Hou et al. demonstrated a multi-component interferometer based on a partially filled dual-core PCF for temperature and strain sensing [11]. In 2017, Lin et al. demonstrated a highly sensitive simultaneous temperature and strain measurement by infiltrating three adjacent air holes of the innermost layer in a PCF [12]. In 2021, Zhou et al. used a section of hybrid-filled PCF to realize the simultaneous measurement of temperature and strain [13].

Unfortunately, the small air holes in PCFs make the selective infiltration of liquid difficult and time-consuming. In recent years, hollow-core anti-resonant fibers (HC-ARFs) have demonstrated their superiority with characteristics such as broad transmission bandwidth and ultra-low loss [14,15]. Since HC-ARFs have a much simpler cladding structure, and the size of the air holes is much larger than those of PCFs, the process of liquid infiltration into the core and micron holes of an HC-ARF is expected to be easier and more efficient. In 2017, Wang et al. have demonstrated experimentally that liquid-filled HC-ARFs are an efficient platform for opto-fluidic applications, such as lasering and sensing [16]. In 2019, Wei et al. numerically investigated the temperature-sensing characteristics of HC-ARFs and found that the temperature sensitivity can reach 1.1 nm/°C when HC-ARFs are fully filled with liquid [17]. In 2021, Han et al. demonstrated a temperature sensor based on HC-ARFs using the SPR effect, and the temperature sensitivity was increased to 2.86 nm/°C [18].

Although HC-ARF is an efficient platform for sensing, the cross-sensitivity of temperature and strain is also inevitable. However, to our best knowledge, dual-parameter sensors for the simultaneous measurement of temperature and strain based on HC-ARFs have not been investigated. In this paper, we work for the first time toward achieving a dual-parameter sensor that can simultaneously measure temperature and strain by designing an HC-ARF with composite nested tubes. Two different sensing mechanisms, the resonance coupling effect and the intermodal interference, are realized in the same section of an HC-ARF fully filled with ethanol. Numerical investigations show that the wavelength of resonant coupling has a sensitivity of 3.36 nm/°C, and the feature wavelength of modal interference has a sensitivity of 0.50 nm/°C in the temperature range of ~20–40 °C. While in the strain range of ~0–4000 με, the wavelength of resonant coupling has a sensitivity of −0.003 nm/με, and the feature wavelength of modal interference has a sensitivity of −0.002 nm/με. The temperature and strain sensor proposed based on the composite nested HC-ARFs has several advantages. First, it is highly sensitive to temperature owing to the large overlap between the fiber modes and the liquid with a high thermos-optic coefficient. Second, compared with dual-parameter sensors based on selectively infiltrated PCFs, the fabrication process of the sensor based on HC-ARFs is much more efficient. As the manufacturing of HC-ARFs becomes mature, dual-parameter sensors based on HC-ARFs with functional liquid will find potential applications in a wide range of fields.

## 2. Principle of the Fiber Sensor

**Figure 1:**
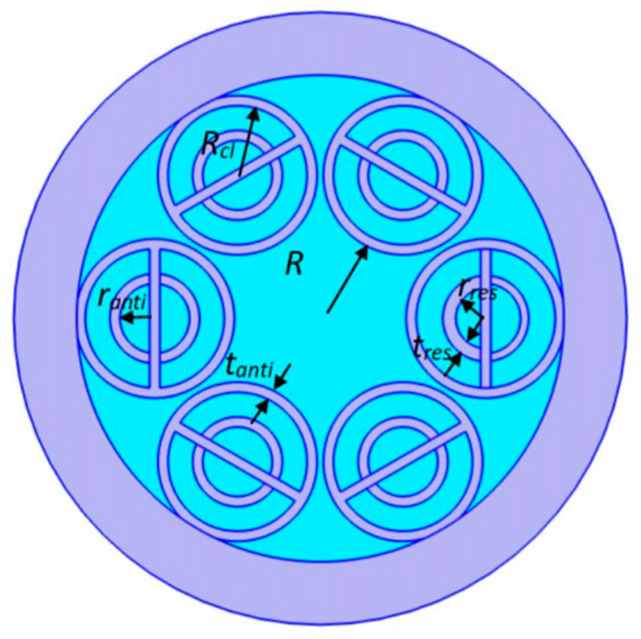
Schematic of the proposed nested HC-ARF.

Figure 1 displays the cross-section of the proposed HC-ARF with air holes that are filled with liquid. The glass is represented by purple regions, and the liquid is represented by blue regions. As shown in Figure 1, six equally spaced outer silica capillaries form the cladding of the fiber, and their radii are all *R_cl_*. The core of the fiber is the area enclosed by the capillaries, with a radius of *R*. Five of the silica capillaries contain conjoined nested anti-resonant tubes, and the radii of the nested anti-resonant tubes are *r_anti_*. The left one contains a hybrid conjoined nested tube, and it consists of a half-circular anti-resonant tube with a radius of *r_anti_* and a half-circular resonant tube with a radius of *r_res_*.

According to the anti-resonant reflective optical waveguide (ARROW) guiding condition [19], we can determine the thickness of the outer capillaries and the anti-resonant tubes as:

(1) $$t_{anti}=\frac{\left(m-0.5\right)\lambda }{2\sqrt{n_{1}^{2}-n_{0}^{2}}},$$

where *λ* is the wavelength, *n*_1_ is the refractive index of silica, *n*_0_ is the refractive index of ethanol, and *m* is a positive-integer order of anti-resonance. When the wavelength is 1.55 µm, *n*_1_ and *n*_0_ are 1.4450 and 1.3605 (the refractive index of ethanol when *T* = 20 °C), and the anti-resonant order *m* is chosen to be 2, we have *t_anti_* = 2.47 µm. Similarly, we can determine the thicknesses of the nested resonant tubes *t_res_* as:

(2) $$t_{res}=\frac{m\lambda }{2\sqrt{n_{1}^{2}-n_{0}^{2}}}$$

The thickness is 3.18 µm using the same parameters.

**Figure 2:**
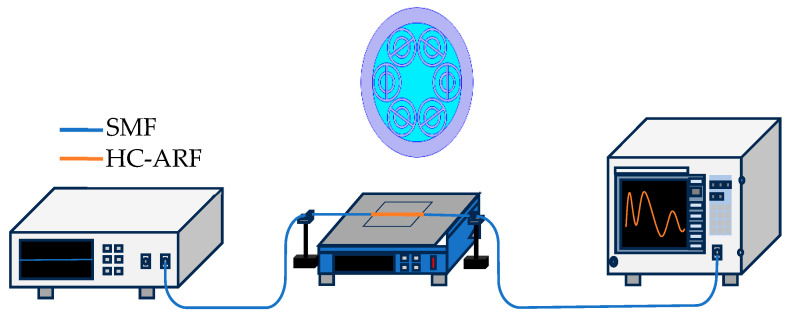
Schematic of the fiber optical temperature and strain sensor based on HC-ARFs.

The schematic of the proposed fiber optical sensor is shown in Figure 2. It consists of an amplified spontaneous emission (ASE) light source, a temperature-controlling platform, strain-controlling clamps, and an optical spectrum analyzer (OSA). Light is incident into the HC-ARF through a section of single-mode fiber, and the transmitted light is detected by the OSA through another section of single-mode fiber.

The fundamental mode field of the single-mode fiber will excite some circular-symmetric modes LP_0m_ in the HC-ARF. The transmission spectrum of the sensor is:

(3) $$\begin{array}{l} I\left(\lambda \right)=I_{0}\left[\sum_{i=1}^{m}\eta_{i}\exp\left(-2\alpha_{i}\left(\lambda \right)\cdot L\right)\right]\\ +2I_{0}\sum_{\begin{array}{l} i,j=1\\ i\neq j \end{array}}^{m}\sqrt{\eta_{i}\eta_{j}}\exp\left(-2\alpha_{i}\left(\lambda \right)\cdot L-2\alpha_{j}\left(\lambda \right)\cdot L\right)\cos\left[\left(\beta_{i}(\lambda )-\beta_{j}(\lambda )\right)\cdot L\right] \end{array}$$

where *L* is the length of the HC-ARF, and *I*_0_ is the incident light intensity. *α*_i,_ *β*_i_ represent the attenuation factor and propagation constant of the LP_0i_ mode, respectively. *η*_i_ represents the power coupling coefficient between the fundamental mode of the single-mode fiber and the LP_0i_ mode of the HC-ARF. *α*_i_ and *β*_i_ can be calculated using the commercial finite-element software COMSOL Multiphysics (v6.1) with a perfectly matched layer (PML) as the boundary condition:

(4) αi=2πImneff_i/λ

(5) βi=2πReneff_i/λ

where Reneff_i and Imneff_i are the real and imaginary parts of the effective refractive index ($n_{eff\_i}$) of each mode, respectively. The confinement loss (CL) of each mode is then defined as:

(6) CLi(dB/m)=20ln(10)⋅2πλImneff_i

The two-mode interference in optical fibers is a widely used mechanism for sensing. However, in single-ring ARFs, the CL of the LP_02_ mode is much higher than that of the LP_01_ mode, and the modal interference effect is not obvious [20]. Due to the leakage property of ARFs, the CL of each core mode is determined by the structure of cladding tubes. In this paper, the CL of the LP_02_ mode is reduced by a proper design of the nested cladding tubes, and an enhanced modal interference effect can be employed to measure the variations in temperature and strain.

**Figure 3:**
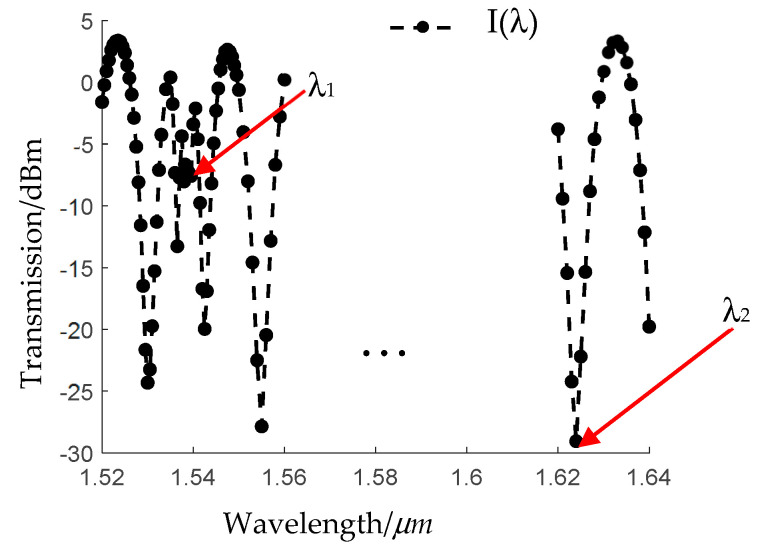
Transmission spectrum of the fiber sensor-based HC−ARFs.

In addition to the modal interference effect, the resonant coupling is also introduced for dual-parameter sensing. When the resonant coupling happens, the LP_02_ mode will couple to the leaky dielectric mode of the nested resonant tubes, leading to an obvious weakening in the output interference pattern. Based on these two sensing mechanisms, the temperature and strain dual-parameter measurement can be achieved if proper feature wavelengths are chosen. Assuming the interference happens mainly between the LP_01_ and LP_02_ mode, and the resonant coupling happens at wavelength *λ*_1_, the transmission spectrum of the sensor is as shown in Figure 3. Since the modal interference and the resonant coupling are both sensitive to variations in temperature and strain, we can choose the wavelength *λ*_1_ in the C-band and the wavelength *λ*_2_ in the U-band as two feature wavelengths. By tracing the two feature wavelengths, the variations of temperature and strain can be demodulated using the cross-sensitive matrix.

However, as can be seen from Figure 3, the feature wavelength *λ*_1_ is not easy to distinguish. The differential spectrum *D* (*λ*) can also be obtained (assuming the variations of the CL of the LP_01_ with wavelength can be neglected):

(7) $$\begin{array}{l} D\left(\lambda \right)=\frac{dI\left(\lambda \right)}{d\lambda }=-2LI_{2}\exp\left(-2\alpha_{2}L\right)\frac{d\alpha_{2}}{d\lambda }\\ -2L\sqrt{I_{1}I_{2}}\exp\left(-\alpha_{1}L-\alpha_{2}L\right)\left\{\cos\left[\left(\beta_{1}-\beta_{2}\right)L\right]\left(\frac{d\alpha_{2}}{d\lambda }\right)+K\sin\left[\left(\beta_{1}-\beta_{2}\right)L\right]\right\} \end{array}$$

**Figure 4:**
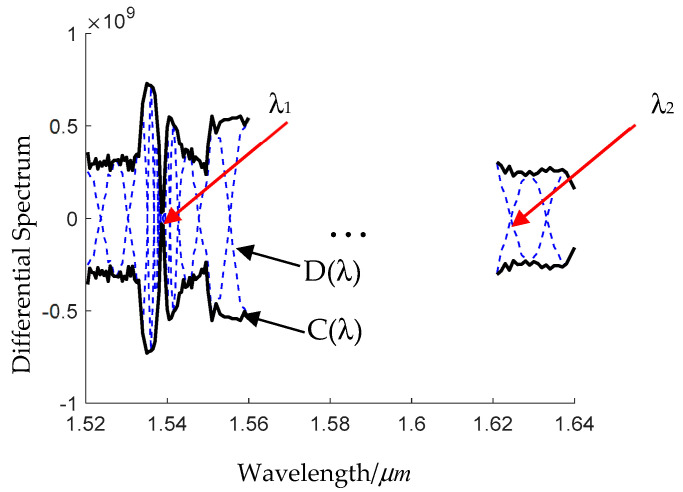
Differential spectrum D (λ) and its envelope C (λ) of the fiber sensor based on HC−ARFs.

The differential spectrum *D* (*λ*) is as shown in Figure 4. In the U-band, the LP_02_ mode is far from the resonant coupling. The attenuation coefficient of the LP_02_ mode changes little with the wavelength ($d\alpha_{2}/d\lambda \approx 0$), and the envelope of the differential spectrum, noted as *C* (*λ*), is a constant. A zero-crossing point in the U-band can be chosen as feature wavelength *λ*_2_. Near the resonant coupling wavelength in the C-band, we have dI/dλ≈0. As a result, the zero-crossing point of the envelope *C* (*λ*) is the feature wavelength *λ*_1_.

Since the transmission characteristics of the proposed fiber are weakly dependent on the polarization state of the incident light, we will focus our investigation on the x-polarization modes in the following. The variation of the polarization state will slightly change the positions of the feature wavelengths, but the sensing characteristics will not be changed.

## 3. Design of the Sensing HC-ARFs

The radius of the core is set to be *R* = 21 µm. Since the core diameter of the HC-ARF is much larger than that of a solid-core single-mode fiber, the higher modes LP_0m_ (m ≠ 1), especially the LP_02_ mode, will be excited. However, there is not a rigorous cut-off condition for modes in the HC-ARF, so the CL of the LP_02_ mode is always much higher than that of the LP_01_ mode. The radii of the conjoined nested resonant tubes *r_anti_* are the main factors affecting the CL of the LP_02_ mode. Therefore, the key parameters of the design of the HC-ARF in this paper are the radii of the conjoined nested resonant tubes in the horizontal direction *r_res_* and the nested anti-resonant tubes *r_anti_*. One is to enhance the effect of the resonant coupling, and the other is to increase the visibility of the modal interference between the LP_01_ mode and LP_02_ mode.

### 3.1. Effect of the Conjoined Nested Anti-Resonant Tube

**Figure 5:**
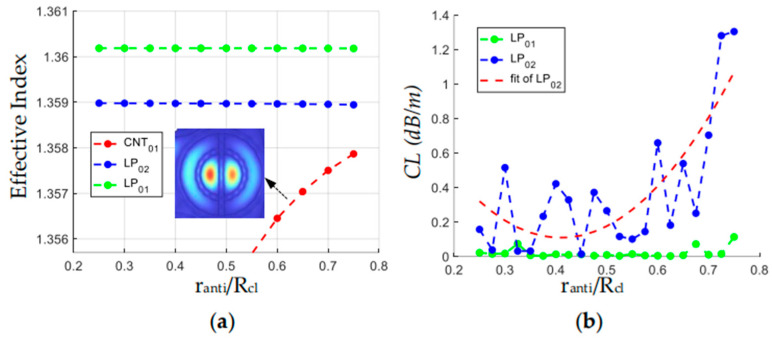
(**a**) Variation of effective refractive indices of LP_01_, LP_02_, and CNT_01_ with *r_anti_*/*R_cl_*, (**b**) Variation of confinement loss of LP_01_ and LP_02_ core modes with *r_anti_*/*R_cl_*.

**Figure 6:**
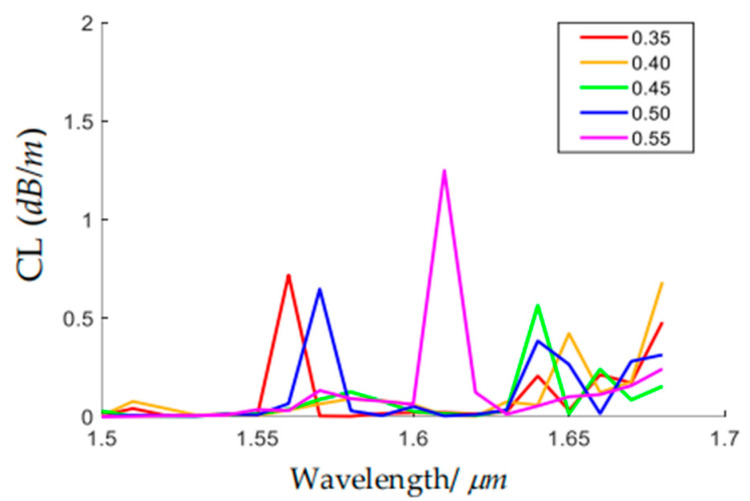
Confinement loss spectrum of LP_02_ core mode in the U-band with different values of *r_anti_*/*R_cl_*.

The HC-ARF with conjoined nested tubes (CNTs) was proposed by Chen in 2018, which can transmit several low-order core modes (LP_01_–LP_31_) with very low confinement loss [21]. The CNTs are regarded as lossy hollow waveguides. When the *n_eff_* of a core mode gets close to that of a CNT mode, a large CL of the core mode will occur due to the energy coupling. When the *n_eff_* of the fundamental tube mode (CNT_01_) is far lower than that of the LP_02_ mode, the CL of the LP_02_ mode will be reduced. We perform a parametric scan of *r_anti_* in the range of 0.25*R_cl_*–0.75*R_cl_*, and the effective refractive indices and CLs for the LP_01_, LP_02,_ and CNT_01_ are calculated, as shown in Figure 5. It can be seen from Figure 5a that the effective refractive index of the CNT_01_, whose electric field is shown in the inset, monotonically increases with *r_anti_*. When it approaches the effective refractive index of the LP_02_ mode, the CL of the LP_02_ increases, as shown in Figure 5b. The LP_02_ mode maintains a stable low CL when *r_anti_* is in the range of 0.35*R_cl_*–0.55 *R_cl_*. Figure 6 depicts the variations of CL of the LP_02_ mode with wavelength when *r_anti_* is set to be from 0.35*R_cl_* to 0.55*R_cl_*. It is shown that near the U-band (~1.60–1.68 μm), the CLs of the LP_02_ mode are all less than 1.4 dB/m. The maximum CL in the U-band is 0.4 dB/m when *r_anti_* is equal to 0.5*R_cl_*, thus *r_anti_* is set to be 0.5*R_cl_* in the following analysis.

### 3.2. Effects of the Nested Resonant Tube

**Figure 7:**
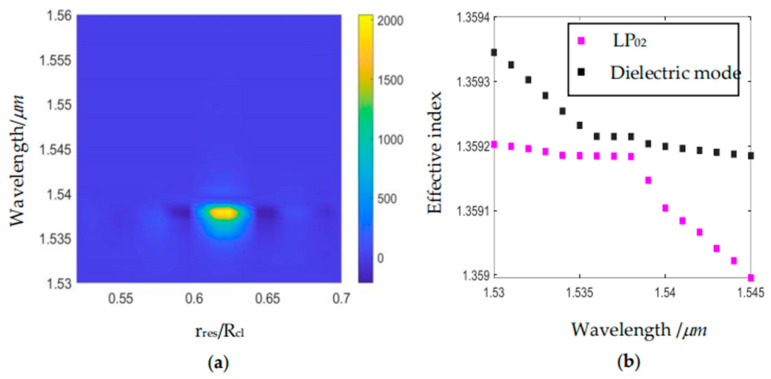
(**a**) The effects of *r_res_*/*R* and wavelength on the confinement loss of LP_02_. (**b**) Variation of the effective refractive indices of LP_02_ and dielectric mode with wavelength.

In order to investigate the effect of the nested resonant tube radius in the horizontal direction, we perform a simultaneous parametric scan for nested resonant tube radii *r_res_* from 0.50*R_cl_* to 0.70*R_cl_* and wavelength *λ* from 1.52 µm to 1.56 µm. The results are shown in Figure 7. Figure 7a shows that the CL of the LP_02_ mode reaches a maximum when the wavelength is 1.538 µm and *r_res_* is 0.62*R_cl_*. Figure 7b depicts that when the nested resonant tube radius *r_res_* equals 0.62*R_cl_,* the effective refractive indices of LP_02_ core mode and the dielectric mode of the nested resonant tube vary with wavelength. The anti-crossing wavelength is 1.538 µm, where the CL of the LP_02_ increases dramatically.

**Figure 8:**
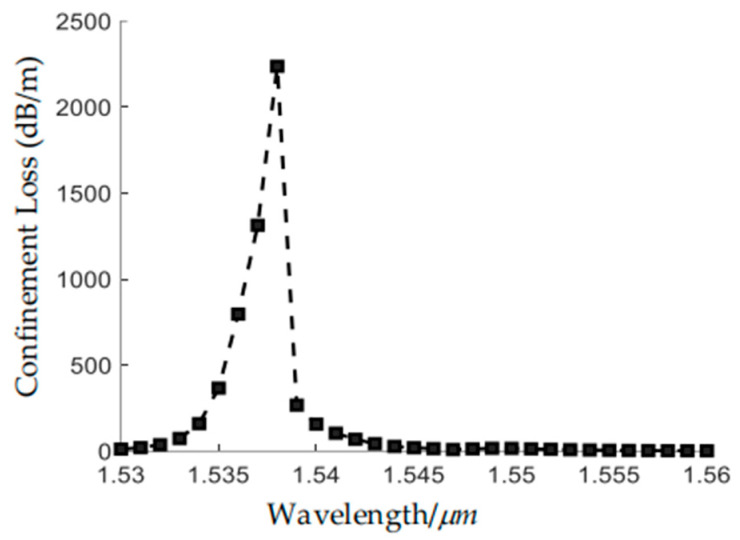
Variation of the confinement loss of LP_02_ mode with wavelength.

Figure 8 shows the variation of the CL of the LP_02_ with a wavelength in the range of 1.53 µm to 1.56 µm. It is shown that the CL reaches its maximum of 2250 dB/m at 1.538 µm.

### 3.3. Sensing Characteristics

The ethanol, which is filled into the HC-ARF, is a thermos-optical liquid. Its refractive index varies linearly with the temperature *T*:

(8) $$n=n_{0}-\alpha_{to}\left(T-T_{0}\right)$$

where *n*_0_ is the refractive index of ethanol at a reference temperature *T*_0_. When *T*_0_ is 20 °C, *n*_0_ = 1.3605. The thermos-optical coefficient of ethanol is *α*_to_ = 3.94 × 10^−4^/°C [22]. Since the thermos-optical coefficient of ethanol is much larger than that of silica (*α*_silica_ = 8.60 × 10^−6^/°C), the thermos-optical effect of silica can be neglected. When the fiber is subject to axial strain, the refractive index will also change due to the elastic-optical effect.

(9) $$\Delta n_{sio_{2}}=-\frac{n_{sio_{2}}^{3}}{2}\left[p_{12}-\nu \left(p_{11}+p_{12}\right)\right]\varepsilon_{z}$$

where $\varepsilon_{z}=\Delta L/L$, and the elastic-optical coefficient of silica is *p*_11_ = 0.12 and *p*_12_ = 0.27. The Possion’s ratio is *ν* = 0.17.

**Figure 9:**
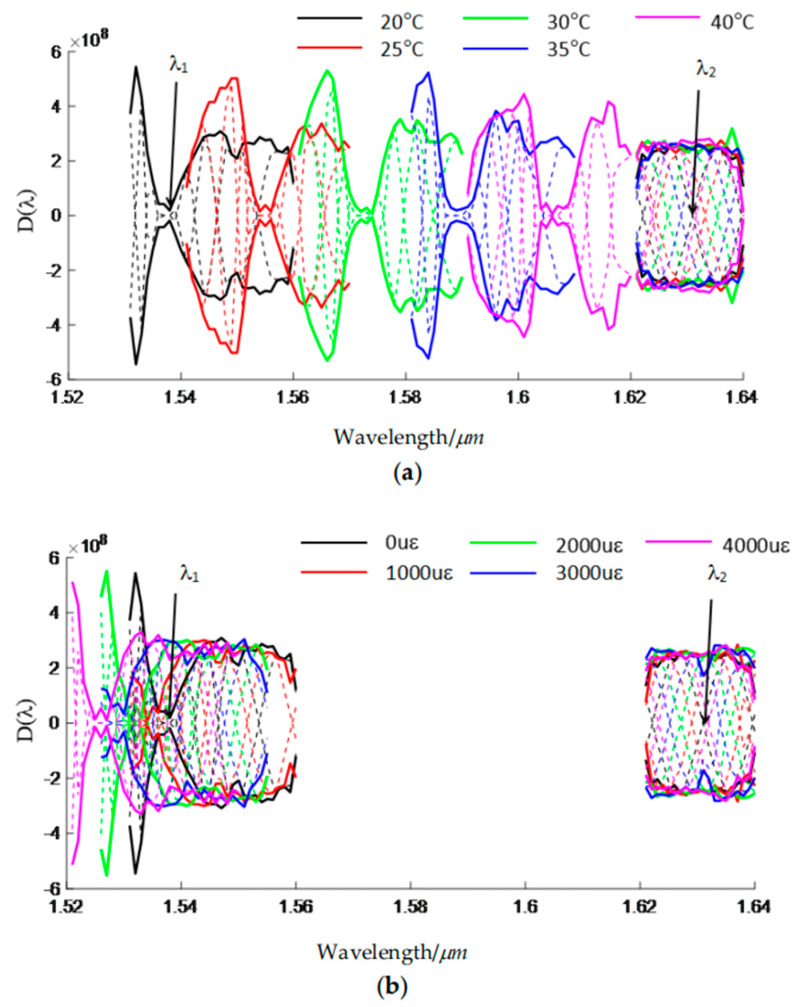
Variation of the differential spectrum and their envelopes with (**a**) temperature and (**b**) strain.

Since the incident field of the sensor is a fundamental mode of a single-mode fiber, it can be obtained that the mainly excited modes are LP_01_ and LP_02_, whose power coupling coefficients are *η*_1_ = 0.5033 and *η*_2_ = 0.2266, respectively. The sum of the coupling coefficients of other higher-order modes is about 0.0635, and the insertion loss of the sensor is, therefore, about 2 dB due to the Fresnel reflection. The differential spectrum *D* (*λ*) can be calculated according to Equation (4). Figure 9 depicts the transmission of the sensor at different temperatures and strains when *r_res_* = 0.62*R_cl_*, *r_anti_* = 0.50*R_cl_*, and *L* = 0.1 m. Figure 9a shows the variation of the transmission spectra with the temperature. It can be seen that the feature wavelength in the C band *λ*_1_ will shift to a longer wavelength when the temperature increases, while the feature wavelength in the U-band *λ*_2_ will also shift to a longer wavelength when the temperature rises. Figure 9b displays the variation of the transmission spectra with the strain. *λ*_1_ and *λ*_2_ both shift to the shorter wavelength when the strain increases.

**Figure 10:**
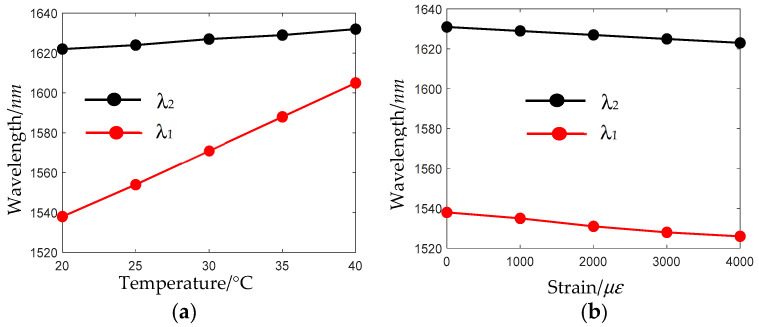
Variation of feature wavelengths of the resonance coupling effect λ_1_ and the intermodal interference λ_2_ with (**a**) temperature and (**b**) strain.

Figure 10 depicts the variation of feature wavelengths *λ*_1_ and *λ*_2_ with temperature and strain, respectively. It can be seen that they both have a good linear response. The sensitivity of *λ*_1_ to temperature and strain are 3.36 nm/°C and −0.003 nm/με, respectively. The sensitivity of *λ*_2_ to temperature and strain are 0.50 nm/°C and −0.002 nm/με, respectively. A quantitative comparison of the performance between the sensors based on composite nested HC-ARFs developed in this study and other reported sensing schemes for simultaneous measurement of strain and temperature is shown in Table 1.

## 4. Discussions

### 4.1. Influence of the Radii of Nested Resonant Tubes

**Figure 11:**
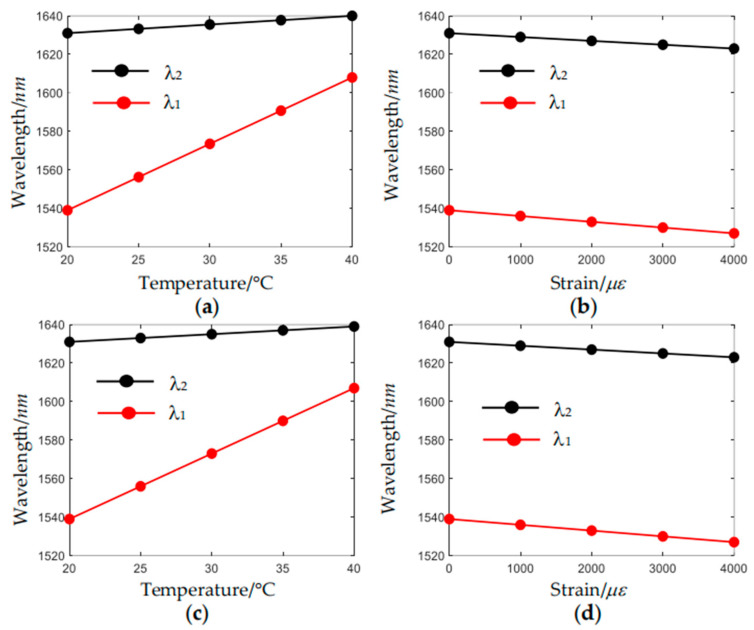
Variation of the feature wavelengths (**a**) with temperature when *r_res_* = 0.60*R_cl_*; (**b**) with strain when *r_res_* = 0.60*R_cl_*, (**c**) with temperature when *r_res_* = 0.64*R_cl_*, (**d**) with strain when *r_res_* = 0.64*R_cl_*.

The radius of the nested resonant tube determines the coupling between the nested dielectric mode and the core mode LP_02_. We chose two cases where *r_res_* = 0.60*R_cl_* and *r_res_* = 0.64*R_cl_* for comparison, and the result is shown in Figure 11. When *r_res_* = 0.60*R_cl_*, the wavelength of resonant coupling has a sensitivity of 3.45 nm/°C, and the feature wavelength of modal interference has a sensitivity of 0.45 nm/°C. In the strain range of ~0–4000 με, the wavelength of resonant coupling has a sensitivity of −0.003 nm/με, and the feature wavelength of modal interference has a sensitivity of −0.002 nm/με. When *r_res_* = 0.64*R_cl_*, the wavelength of resonant coupling has a sensitivity of 3.40 nm/°C, and the feature wavelength of modal interference has a sensitivity of 0.40 nm/°C. In the strain range of ~0–4000 με, the wavelength of resonant coupling has a sensitivity of −0.003 nm/με, and the feature wavelength of modal interference has a sensitivity of −0.002 nm/με. Since the effective refractive indices of the LP_01_ and LP_02_ change little with the radii of nested resonant tubes, the sensitivities of *λ*_2_ to temperature and strain do not change with the radii of nested resonant tubes. The effective refractive indices of the dielectric mode will change slightly with the radii. Thus, there are small changes in the sensitivities of *λ*_1_ to temperature and strain.

### 4.2. Influence of the Thickness of Resonant Tubes

**Figure 12:**
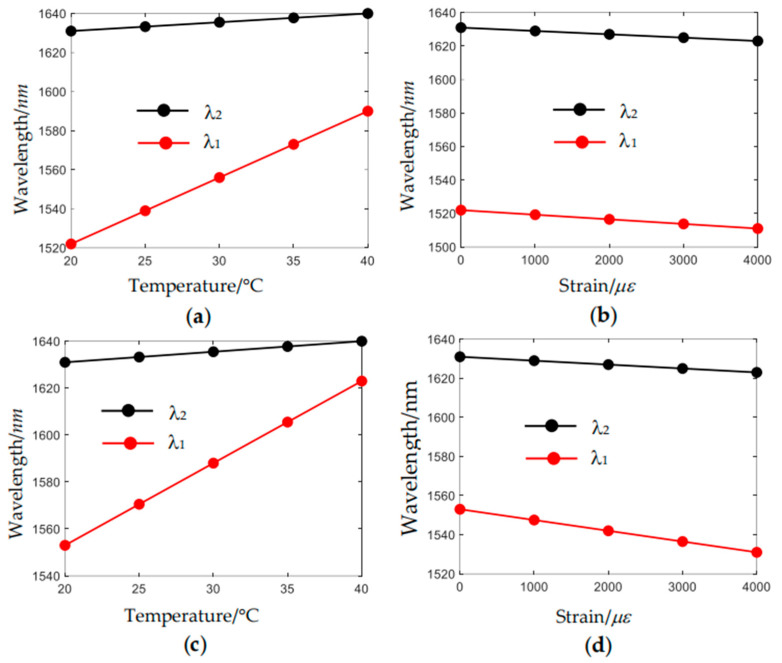
Variation of the feature wavelengths (**a**) with temperature when *t_anti_* = 3.15 μm; (**b**) with strain when *t_anti_* = 3.15 μm; (**c**) with temperature when *t_anti_* = 3.21 μm; (**d**) with strain when *t_anti_* = 3.21 μm.

In the fabrication process, the resonant tube thickness may deviate from the expected value of 3.18 µm. Figure 12 depicts the sensing characteristics of the sensor when the resonant tube thickness is set to be 3.15 µm and 3.21 µm, respectively. It can be seen in Figure 12 that the resonant wavelength *λ*_1_ shifts to a longer wavelength when the thickness of the resonant tube increases. When the thickness is 3.15 µm, the wavelength of resonant coupling has a sensitivity of 3.40 nm/°C, and the feature wavelengths of modal interference have a sensitivity of 0.50 nm/°C. While in the strain range of ~0–4000 με, the wavelength of resonant coupling has a sensitivity of −0.003 nm/με, and the feature wavelengths of modal interference have a sensitivity of −0.002 nm/με. When the thickness is 3.21 µm, the wavelength of resonant coupling has a sensitivity of 3.50 nm/°C, and the feature wavelengths of modal interference have a sensitivity of 0.50 nm/°C. The wavelength of resonant coupling has a sensitivity of −0.003 nm/με, and the feature wavelengths of modal interference have a sensitivity of −0.002 nm/με.

### 4.3. Influence of Polarization States

**Figure 13:**
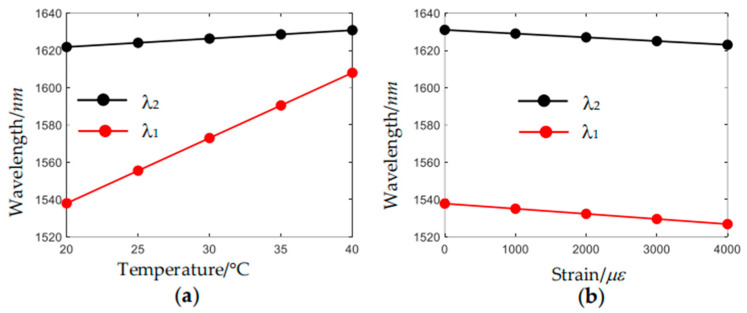
Sensing characteristics of the y-polarized light when *r_res_* = 0.62*R_cl_* and *t_anti_* = 3.18 μm. (**a**) Variation of the feature wavelengths with temperature; (**b**) variation of the feature wavelengths with strain.

In the above structure simulation, the input light is set to be x-polarized. Here, we investigate whether the polarization state affects the sensing characteristics. The sensing characteristic of the y-polarized light is shown in Figure 13. Numerical simulation results indicate that in the temperature range of ~20–40 °C, the wavelength of resonant coupling has a sensitivity of 3.50 nm/°C, and the feature wavelengths of modal interference have a sensitivity of 0.45 nm/°C. While in the strain range of ~0–4000 με, the wavelength of resonant coupling has a sensitivity of −0.003 nm/με, and the feature wavelengths of modal interference have a sensitivity of −0.002 nm/με. Therefore, the state of polarization has a weak influence on the sensitivities of the resonant wavelength *λ*_1_ but has no influence on the sensitivities of the feature wavelength of modal interference *λ*_2_.

The above numerical investigations show that the proposed temperature and strain sensor based on composite nested HC-ARFs has higher sensitivities of temperature and strain compared to sensing devices based on solid-core fibers. Compared to sensor devices based on PCFs with selective infiltration, the fabrication process of the sensor based on HC-ARF is much more efficient due to the full infiltration of the microchannels with larger microchannels. The sensitivities are lower than some schemes based on PCFs. There are two reasons for this drawback: (1) the flat dispersion characteristics of core modes of HC-ARFs; (2) the reduction of the proportion of stress-sensitive material in HC-ARFs. Nevertheless, the fluctuation in the sensing characteristics caused by the fiber fabrication errors is also reduced.

## 5. Conclusions

A highly sensitive fiber sensor for simultaneous measurement of temperature and strain-based HC-ARFs has been proposed. The HC-ARFs consist of two kinds of nested tubes. Five conjoined nested anti-resonant tubes are introduced to suppress the confinement loss of the higher-order mode LP_02_. The left one is the hybrid conjoined nested tube, and it consists of a half-circular anti-resonant tube and a half-circular resonant tube. Numerical simulation results indicate that the sensitivities of the resonant coupling wavelength to temperature and strain are 3.36 nm/°C and −0.003 nm/με. The sensitivities of feature wavelength of modal interference to temperature and strain are 0.50 nm/°C and −0.002 nm/με. The influence of the structural parameters of the HC-ARF on the sensing characteristics is also discussed. Since no special post-processing is needed, such as selective infiltration or material coating for the sensors proposed, the sensor proposed provides a promising mechanism for the simultaneous measurement of temperature and strain.

## Figures and Tables

**Table 1:** Quantitative comparison of performance of the reported schemes for simultaneous measurement of temperature and strain.

Ref.	Sensing Scheme	Temperature Sensitivity	Strain Sensitivity
[4]	FBG pair	−6.75 pm/°C; 7.25 pm/°C	0.79 pm/με; 0.80 pm/με
[5]	LPG pair	30 pm/°C; 500 pm/°C	0; 0.8 pm/με
[6]	FBG with FLM	85 pm/°C; 550 pm/°C	−4.37 pm/με; 29.84 pm/με
[7]	FBG with MZI	14.8 pm/°C; 43.5 pm/°C	1.49 pm/με; −2.58 pm/με
[8]	Cascaded SI	6.13 pm/°C; 6.08 pm/°C	−124.2 pm/με; 122.1 pm/με
[9]	Dual MZI	49.4 pm/°C; 43.3 pm/°C	−0.3 pm/με; −2.0 pm/με
[10]	double-filled PCF	42.8 nm/°C; −11.3 nm/°C	38.0 pm/με; 8.7 pm/με
[11]	partial-filled DCF	5.4 nm/°C; 12 pm/°C	−2.0 pm/με; −2.1 pm/με
[12]	triple-filled PCF	14.7 nm/°C; 6.6 nm/°C	12.6 pm/με; 13.0 pm/με
[13]	hybrid-filled PCF	14.1 nm/°C; −5.6 nm/°C	35.0 pm/με; 31.1 pm/με
This work	Composite nested HC-ARF	3.36 nm/°C; 0.5 nm/°C	−3.0 pm/με; −2.0 pm/με

## Data Availability

Data are contained within the article.
